# Supplementary material for: Comparative Efficacy and Safety of Anti-PD-1/PD-L1 Immune Checkpoint Inhibitors for Refractory or Relapsed Advanced Non-Small-Cell Lung Cancer—A Systematic Review and Network Meta-Analysis
Source: Cancers (Basel). 2020 Dec 27;13(1):52. doi: 10.3390/cancers13010052 (PMC7796092; doi:10.3390/cancers13010052)
Supplement: Supplementary file 1 [file cancers-13-00052-s001.pdf]

Supplementary information

Figure S1: A scatter diagram of the SUCRA, of the efficacy in terms of PFS and safety in terms of G3–5AEs, of the four therapeutic regimens

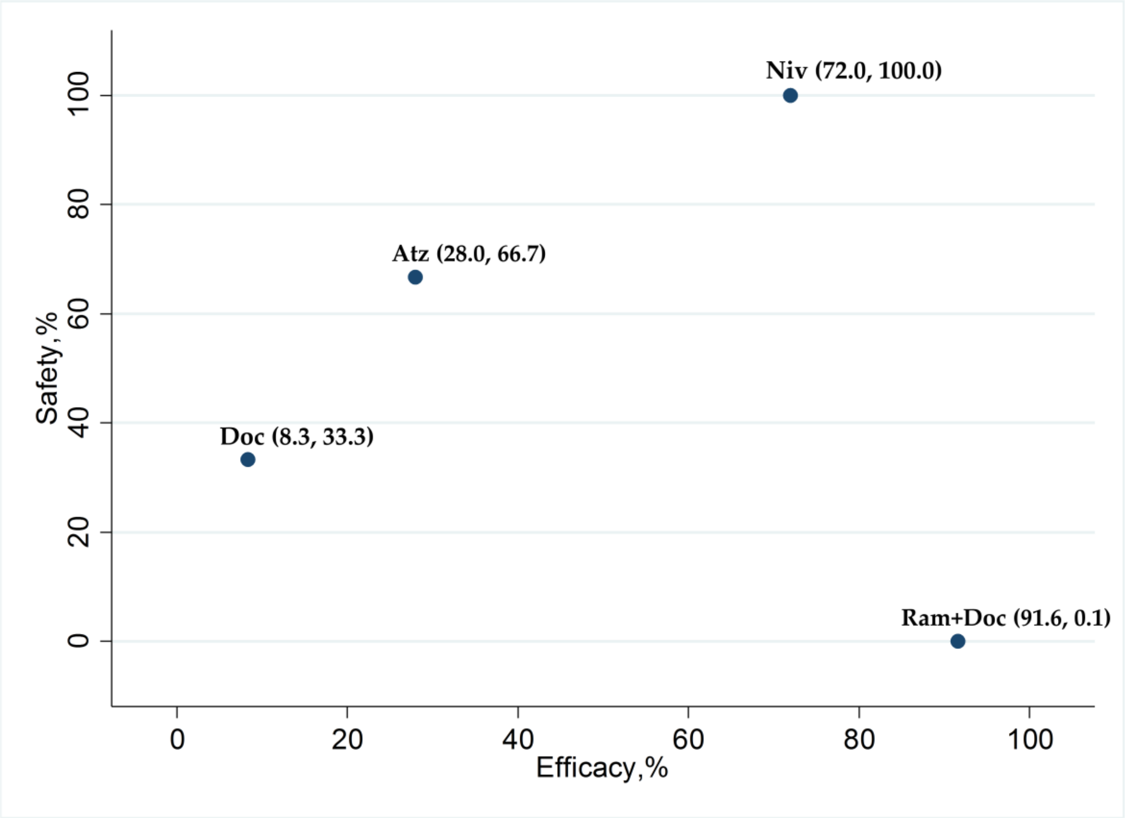

Figure S1. Scatter diagram of the surface under the cumulative ranking curve (SUCRA) corresponding to efficacy in progression-free survival and safety in grade 3–5 adverse events (G3–5AEs) of four therapeutic regimens, Ram+Doc, Doc, Niv, and Atz, in patients with refractory or relapsed advanced NSCLC. Data are presented as SUCRA in OS and SUCRA in G3–5AEs in each plot of the four therapeutic regimens. In terms of efficacy, Ram+Doc treatment ranked the highest and Niv was second, followed by Atz, and finally Doc. Niv treatment ranked highest in safety, followed by Atz, Doc, and finally Ram+Doc; Niv (nivolumab); Atz, (atezolizumab); Ram+Doc (ramucirumab plus docetaxel); Doc (docetaxel).

Table S1: Sensitivity analysis

Treatment comparisons	HR[95%CrI]
Ram+Doc vs. Doc	0.862 [0.752-0.983]
Niv vs. Doc	0.683 [0575-0.806]
Atz vs. Doc	0.713[0.587-0.856]
Niv vs. Ram+Doc	0.796[0.639-0.982]
Atz vs. Ram+Doc	0.831[0.655-1.040]
Atz vs. Niv	1.051[0.809-1.342]

A sensitivity analysis was performed by excluding patients who had received two previous systemic anti-cancer treatments in the OAK study. Data are expressed as hazard ratio (HR) for overall survival (OS) and 95% credible intervals (CrI); Ram+Doc (ramucirumab plus docetaxel); Doc (docetaxel); Niv (nivolumab); Atz, (atezolizumab).

Table S2: Sensitivity analysis for ranking assessment

Treatment regimens	SUCRA [rank]
Doc	0.5 [4]
Ram+Doc	35.2 [3]
Niv	87.0 [1]
Atz	77.4 [2]

A sensitivity analysis was performed by excluding patients who had received two previous systemic anti-cancer treatments in the OAK study. Data are expressed as surface under the cumulative ranking curve (SUCRA) for overall survival (OS) and efficacy rank within each treatment regimen; Ram+Doc, (ramucirumab plus docetaxel); Doc (docetaxel); Niv (nivolumab); Atz (atezolizumab).

Figure S2: Risk of bias summery

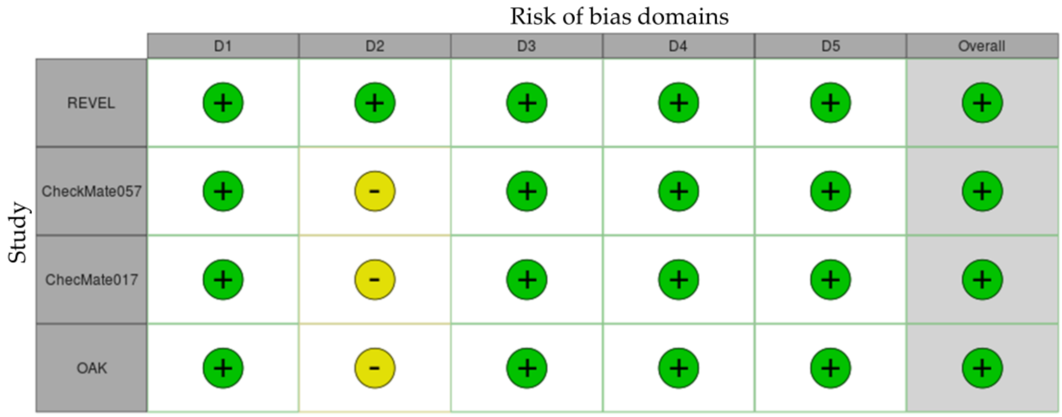

Domains:
D1: Bias arising from the randomization process
D2: Bias due to deviations from intended interventions
D3: Bias due to missing outcome data
D4: Bias in measurement of the outcome
D5: Bias in selection of the reported result

Figure S2. Risk of bias summary. The risk of bias summary involved a review of authors' judgments regarding each risk of bias item for each included study. The symbols "+," "−", and "×" indicate a low risk of bias, some concerns, and a high risk of bias, respectively. The quality of the included studies was good because none of the studies was considered as high risk of bias, although three had some concerns for "bias due to deviations from intended interventions," as these studies were open-label.

Table S3. Key inclusion criteria of LUME-Lung 1 and KEYNOTE-010

Study names	Key inclusion criteria
LUME-Lung 1	・18 years of age or older<br>・histology or cytology confirmed stage IIIB or IV recurrent NSCLC<br>・progressed during or after a single platinum-based chemotherapy regimen<br>・Performance status range of 0 or 1
KEYNOTE-010	・18 years of age or older<br>・histology or cytology confirmed stage IIIB or IV recurrent NSCLC whose tumors express PD-L1<br>・progressed during or after at least a platinum-containing systemic therapy<br>・Performance status range of 0 or 1

NSCLC, non-small-cell lung cancer

Table S4. Main characteristic of LUME-Lung 1 and KEYNOTE-010

Study names	Treatment arms	N	Age-yr median(IQR)	Female Sex No. (%)	ECOG PS No. (%)	Histlogic type No. (%)
LUME-Lung1	Nintedanib 200 mg twice daily on day 2-21 plus Docetaxel 75 mg/m2 on day 1, e3w	655	60(53-67)	179(27.3)	PS0: 187(28.5)<br>PS1: 467(71.3)	Squamous: 276 (42.1)<br>Adeno: 322 (49.2)<br>Large: 25 (3.8)<br>Combination: 4 (0.6)<br>Other: 28 (4.3)

	placebo twice daily on day 2-21<br>plus Docetaxel 75 mg/m2 on day 1, e3w	659	60(54-66)	180(27.3)	PS0: 189(28.7)<br>PS1: 470(71.3)	Squamous:279 (42.3)<br>Adeno: 336 (51.0)<br>Large: 16 (2.4)<br>Combination: 5 (0.8)<br>Other: 23 (3.5)
		**total, 1314**				
KEYNOTE-010	Pembrolizumab 2 mg/kg e3w	344	63.0 (56.0-69.0)	132(38)	PS0: 112 (33)<br>PS1: 229 (67)<br>PS2: 3 (1)<br>PS3: 0 (0)<br>unknown: 0 (0)	squamous: 76 (22)<br>non-squamous: 240 (70)<br>other: 9 (3)<br>unknown:19 (6)
	Pembrolizumab 10 mg/kg e3w	346	63.0(56.0-69.0)	133(38)	PS0: 120 (35)<br>PS1: 225 (65)<br>PS2: 1 (<1)<br>PS3: 0 (0)<br>unknown: 0 (0)	squamous: 80 (23)<br>non-squamous:244 (71)<br>other: 6 (2)<br>unknown: 16 (5)
	Docetaxel 75 mg/m2 e3w	343	62.0 (56.0-69.0)	134(39)	PS0: 116 (34)<br>PS1: 224 (65)	squamous: 66 (19)<br>non-squamous: 240 (70)

	PS2: 1 (<1)	other: 10 (3)
	PS3: 1 (<1)	unknown: 27 (8)
	unknown: 1 (<1)	
**total, 1033**		

N, sample size; ECOG, Eastern Cooperative Oncology Group; PS, performance status; e3w, every 3 weeks.

Figure S3. Risk of bias summery of LUME-Lung 1 and KEYNOTE-010

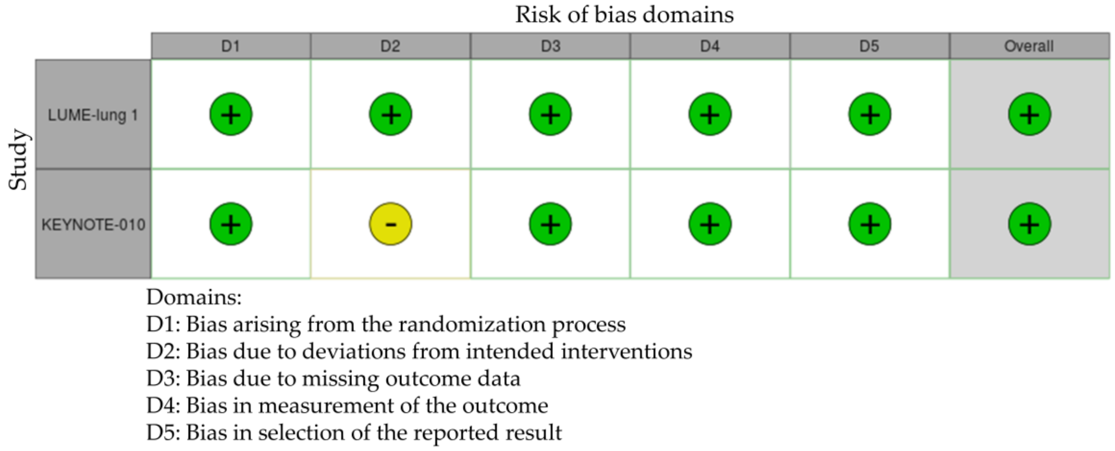

Figure S3. Risk of bias summary. The risk of bias summary involved a review of authors' judgments regarding each risk of bias item for each study. The symbols "+," "−", and "×" indicate a low risk of bias, some concerns, and a high risk of bias, respectively. The quality of the included studies was good because none of the studies was considered high risk of bias, although one of two had some concerns for "bias due to deviations from intended interventions," as these studies were open-label.

Table S5. The results of the OS comparison (six RCTs of REVEL, CheckMate057, CheckMate017, OAK, LUME-Lung 1, KEYNOTE-010 were included)

Treatment comparisons	HR [95% CrI]
Ram+Doc vs. Doc	0.862 [0.752–0.983]
Niv vs. Doc	0.683 [0.575–0.805]
Atz vs. Doc	0.733 [0.616–0.865]
Pem2 vs. Doc	0.714 [0.577–0.875]
Pem10 vs. Doc	0.614 [0.493–0.755]
Nin+Doc vs. Doc	0.942 [0.836–1.057]
Niv vs. Ram+Doc	0.796 [0.638–0.983]
Atz vs. Ram+Doc	0.854 [0.684–1.054]

Pem2 vs. Ram+Doc	0.833 [0.644–1.059]
Pem10 vs. Ram+Doc	0.716 [0.552–0.913]
Nin+Doc vs. Ram+Doc	1.098 [0.915–1.306]
Atz vs. Niv	1.080 [0.844–1.363]
Pem2 vs. Niv	1.053 [0.798–1.365]
Pem10 vs. Niv	0.905 [0.683–1.176]
Nin+Doc vs. Niv	1.389 [1.125–1.696]
Pem2 vs. Atz	0.982 [0.744–1.273]
Pem10 vs. Atz	0.844 [0.637–1.098]
Nin+Doc vs. Atz	1.295 [1.048–1.583]
Pem10 vs. Pem2	0.869 [0.638–1.158]
Nin+Doc vs. Pem2	1.333 [1.041–1.681]
Nin+Doc vs. Pem10	1.552 [1.208–1.961]

Comparative overall survival (OS) of patients with refractory or relapsed advanced non-small-cell lung cancer in the Doc, Niv, Atz, Pen2, Pem10, and Nin+Doc groups from six RCTs; REVEL, CheckMate057, CheckMate017, OAK, LUME-Lung 1, and KEYNOTE-010. Only PD-L1-positive (≥1%) patients were included in KEYNOTE-010, but no restrictions on PD-L1 status were placed on the patient inclusion criteria for the other RCTs. It should be noted that there is heterogeneity regarding PD-L1 status among the RCTs included in this analysis. Comparisons are represented as treatment A versus treatment B. Data are presented as hazard ratios (HR) with 95% credible intervals (CrI); Doc (docetaxel); Niv (nivolumab) ; Atz (atezolizumab); Pem2 (pembrolizumab 2mg /kg); Pem10, (pembrolizumab 10 mg /kg); Nin+Doc (nintedanib plus docetaxel).

Table S6. Results of subgroup analysis for non-squamous OS comparisons (five RCTs of REVEL, CheckMate057, OAK, LUME-Lung 1, and KEYNOTE-010 were included).

Treatment comparisons	HR [95% CrI]
Ram+Doc vs. Doc	0.833 [0.708–0.972]
Niv vs. Doc	0.734 [0.595–0.897]
Atz vs. Doc	0.734 [0.599–0.890]
Pem (pooled) vs. Doc	0.634 [0.501–0.792]
Nin+Doc vs. Doc	0.834 [0.698–0.987]
Niv vs. Ram+Doc	0.888 [0.680–1.142]
Atz vs. Ram+Doc	0.887 [0.682–1.134]
Pem (pooled) vs. Ram+Doc	0.767 [0.575–1.004]
Nin+Doc vs. Ram+Doc	1.008 [0.792–1.264]
Atz vs. Niv	1.011 [0.751–1.330]
Pem (pooled) vs. Niv	0.874 [0.635–1.173]

Nin+Doc vs. Niv	1.148 [0.869–1.487]
Pem (pooled) vs. Atz	0.874 [0.637–1.167]
Nin+Doc vs. Atz	1.148 [0.873–1.481]
Nin+Doc vs. Pem (pooled)	1.332 [0.987–1.754]

Comparative overall survival (OS) of subgroup with refractory or relapsed advanced non-squamous non-small-cell lung cancer in the Doc, Niv, Atz, Pem (pooled) groups from five RCTs; REVEL, CheckMate057, OAK, LUME-Lung 1, and KEYNOTE-010. Only PD-L1-positive (≥1%) patients were included in KEYNOTE-010, but no restrictions on PD-L1 status were placed on the patient inclusion criteria for the other RCTs. Thus, it should be noted that there is heterogeneity regarding PD-L1 status among the RCTs included in this analysis. Comparisons are represented as treatment A versus treatment B. Data are presented as hazard ratios (HR) with 95% credible intervals (CrI); Doc (docetaxel); Niv (nivolumab); Atz (atezolizumab); Pem pooled (pembrolizumab 2 mg /kg, or 10 mg /kg); Nin+Doc (nintedanib plus docetaxel).

Table S7. Results of subgroup analysis for squamous OS comparisons (four RCTs of REVEL, CheckMate017, OAK, KEYNOTE-010 were included)

Treatment comparisons	HR [95% CrI]
Ram+Doc vs. Doc	0.890 [0.692–1.130]
Niv vs. Doc	0.597 [0.440–0.790]
Atz vs. Doc	0.738 [0.541–0.983]
Pem (pooled) vs. Doc	0.755 [0.501–1.090]
Niv vs. Ram+Doc	0.681 [0.455–0.977]
Atz vs. Ram+Doc	0.842 [0.561–1.216]
Pem (pooled) vs. Ram+Doc	0.861 [0.529–1.330]
Atz vs. Niv	1.265 [0.815–1.881]
Pem (pooled) vs. Niv	1.293 [0.771–2.036]
Pem (pooled) vs. Atz	1.047 [0.621–1.659]

Comparative overall survival (OS) of subgroup with refractory or relapsed advanced squamous non-small-cell lung cancer in the Doc, Niv, Atz, Pem (pooled) groups from four RCTs; REVEL, OAK, and KEYNOTE-010. Only PD-L1-positive (≥1%) patients were included in KEYNOTE-010, but no restrictions on PD-L1 status were placed on the patient inclusion criteria for the other RCTs. Thus, it should be noted that there is heterogeneity regarding PD-L1 status among the RCTs included in this analysis. Comparisons are represented as treatment A versus treatment B. Data are presented as hazard ratios (HR) with 95% credible intervals (CrI); Doc (docetaxel); Niv (nivolumab); Atz (atezolizumab); Pem pooled (pembrolizumab 2mg /kg, or 10mg /kg).

Table S8. The results of the PFS comparison (six RCTs REVEL, CheckMate057, CheckMate017, OAK, LUME-Lung 1, and KEYNOTE-010, were included).

Treatment comparisons	HR [95% CrI]
Ram+Doc vs. Doc	0.761 [0.675–0.855]
Niv vs. Doc	0.816 [0.699–0.947]
Atz vs. Doc	0.953 [0.820–1.100]
Pem2 vs. Doc	0.884 [0.739–1.049]
Pem10 vs. Doc	0.793 [0.662–0.944]
Nin+Doc vs. Doc	0.792 [0.679–0.918]
Niv vs. Ram+Doc	1.076 [0.884–1.299]
Atz vs. Ram+Doc	1.256 [1.035–1.509]
Pem2 vs. Ram+Doc	1.165 [0.938–1.432]
Pem10 vs. Ram+Doc	1.046 [0.841–1.287]
Nin+Doc vs. Ram+Doc	1.044 [0.859–1.259]
Atz vs. Niv	1.174 [0.944–1.443]
Pem2 vs. Niv	1.089 [0.857–1.364]
Pem10 vs. Niv	0.978 [0.768–1.226]
Nin+Doc vs. Niv	0.976 [0.783–1.202]
Pem2 vs. Atz	0.933 [0.737–1.164]
Pem10 vs. Atz	0.838 [0.661–1.047]
Nin+Doc vs. Atz	0.836 [0.674–1.026]
Pem10 vs. Pem2	0.905 [0.700–1.152]
Nin+Doc vs. Pem2	0.904 [0.712–1.131]
Nin+Doc vs. Pem10	1.007 [0.792–1.259]

Comparative progression free survival (PFS) of patients with refractory or relapsed advanced non-small-cell lung cancer in the Doc, Niv, Atz, Pem2, Pem10, and Nin+Doc groups from six RCTs; REVEL, CheckMate057, CheckMate017, OAK, LUME-Lung 1, and KEYNOTE-010. Only PD-L1-positive (≥1%) patients were included in KEYNOTE-010, but no restrictions on PD-L1 status were placed on the patient inclusion criteria for the other RCTs. Thus, it should be noted that there is heterogeneity regarding PD-L1 status among the RCTs included in this analysis. Comparisons are represented as treatment A versus treatment B. Data are presented as hazard ratios (HR) with 95% credible intervals (CrI); Doc (docetaxel); Niv (nivolumab); Atz (atezolizumab); Pem2 (pembrolizumab 2mg /kg); Pem10, (pembrolizumab 10mg /kg); Nin+Doc (nintedanib plus docetaxel).

Table S9. Results of the comparison of G3-5AEs incidences (six RCTs of REVEL, CheckMate057, CheckMate017, OAK, LUME-Lung 1, KEYNOTE-010 were included).

Treatment comparisons	RR [95% CrI]
Ram+Doc vs. Doc	1.100 [1.032–1.172]
Niv vs. Doc	0.175 [0.127–0.235]
Atz vs. Doc	0.342 [0.274–0.421]
Pem2 vs. Doc	0.365 [0.263–0.495]
Pem10 vs. Doc	0.455 [0.339–0.599]
Nin+Doc vs. Doc	1.111 [1.028–1.198]
Niv vs. Ram+Doc	0.159 [0.115–0.215]
Atz vs. Ram+Doc	0.311 [0.247–0.386]
Pem2 vs. Ram+Doc	0.332 [0.237–0.453]
Pem10 vs. Ram+Doc	0.414 [0.306–0.548]
Nin+Doc vs. Ram+Doc	1.010 [0.914–1.115]
Atz vs. Niv	2.006 [1.351–2.871]
Pem2 vs. Niv	2.140 [1.341–3.247]
Pem10 vs. Niv	2.668 [1.712–3.963]
Nin+Doc vs. Niv	6.515 [4.680–8.833]
Pem2 vs. Atz	1.080 [0.723–1.553]
Pem10 vs. Atz	1.346 [0.928–1.892]
Nin+Doc vs. Atz	3.287 [2.601–4.100]
Pem10 vs. Pem2	1.280 [0.816–1.914]
Nin+Doc vs. Pem2	3.125 [2.224–4.269]
Nin+Doc vs. Pem10	2.494 [1.837–3.304]

Comparative safety for incidence of grade 3-5 drug related adverse events (G3-5AEs) in patients with refractory or relapsed advanced non-small-cell lung cancer in the Doc, Niv, Atz, Pem2, Pem10, and Nin+Doc groups from six RCTs: REVEL, CheckMate057, CheckMate017, OAK, LUME-Lung 1, and KEYNOTE-010. Only PD-L1-positive (≥1%) patients were included in KEYNOTE-010, but no restrictions on PD-L1 status were placed on the patient inclusion criteria for the other RCTs. It should be noted that there is heterogeneity regarding PD-L1 status among the RCTs included in this analysis. Comparisons are represented as treatment A versus treatment B. Data are presented as risk ratios (RR) with 95% credible intervals (CrI); Doc (docetaxel); Niv (nivolumab); Atz (atezolizumab); Pem2, (pembrolizumab 2mg /kg); Pem10 (pembrolizumab 10mg /kg); Nin+Doc (nintedanib plus docetaxel).

Table S10. The results of SUCRA values (rank) for efficacy and safety outcomes (six RCTs REVEL, CheckMate057, CheckMate017, OAK, LUME-Lung 1, KEYNOTE-010, were included).

	OS	OS non-sq	OS sq	PFS	G3-5AEs
Doc	2.8 (7)	0.6 (6)	6.1 (5)	5.5 (7)	33.2 (5)
Ram+Doc	33.0 (5)	36.9 (5)	31.2 (4)	82.6 (1)	9.5 (6)
Niv	75.6 (2)	66.6 (3)	91.0 (1)	61.7 (3)	100.0 (1)
Atz	62.1 (4)	66.8 (2)	62.4 (2)	20.0 (6)	75.9 (2)
Pem2	67.3 (3)	NE	NE	39.7 (5)	70.6 (3)
Pem10	92.1 (1)	NE	NE	70.0 (4)	53.5 (4)
Pem (pooled)	NE	92.0 (1)	59.3 (3)	NE	NE
Nin+Doc	17.2 (6)	37.1 (4)	NE	70.6 (2)	7.2 (7)

The surface under the cumulative ranking curve (SUCRA) for the efficacy in terms of overall survival (OS), subgroup analysis of OS by histological type (non-squamous or squamous), progression-free survival (PFS), and safety in terms of ≥ grade 3 adverse events (G3-AEs) in patients with refractory or relapsed NSCLC. Data are listed as SUCRA values with (rank). Doc (docetaxel); Niv (nivolumab); Atz, (atezolizumab); Pem2 (pembrolizumab 2 mg /kg); Pem10 (pembrolizumab 10 mg /kg); Pem pooled; (pembrolizumab 2 mg /kg, or 10 mg /kg); Nin+Doc (nintedanib plus docetaxel).

Table S11. Results of OS comparison in PD-L1-positive non-squamous subgroup (two RCTs of CheckMate057 and KEYNOTE-010 were included).

Treatment comparisons	HR [95% CrI]
Niv vs. Doc	0.598 [0.427–0.814]
Pem (pooled) vs Doc	0.634 [0.500–0.792]
Pem (pooled) vs Niv	1.090 [0.720–1.588]

Comparative overall survival (OS) of subgroup with programmed cell death legend 1 (PD-L1) positive (≥1 %) refractory or relapsed advanced non-squamous non-small-cell lung cancer in the Doc, Niv, and Pem (pooled) groups from two RCTs; CheckMate057 and KEYNOTE-010. Comparisons are represented as treatment A versus treatment B. Data are presented as hazard ratios (HR) with 95% credible intervals (CrI); Doc (docetaxel); Niv (nivolumab); Pem pooled (pembrolizumab 2mg /kg, or 10mg /kg).

Table S12. Results of OS comparison in PD-L1-positive squamous subgroup (two RCTs of CheckMate017 and KEYNOTE-010 were included.

Treatment comparisons	HR [95% CrI]
Niv vs. Doc	0.708 [0.441–1.079]
Pem (pooled) vs Doc	0.754 [0.500–1.092]
Pem (pooled) vs Niv	1.123 [0.593–1.945]

Comparative overall survival (OS) of subgroup with programmed cell death legend 1 (PD-L1) positive (≥1 %) refractory or relapsed advanced squamous non-small-cell lung cancer in the Doc, Niv, Pem (pooled) groups from two RCTs; CheckMate017 and KEYNOTE-01. Comparisons are represented as treatment A versus treatment B. Data are presented as hazard ratios (HR) with 95% credible intervals (CrI); Doc (docetaxel); Niv (nivolumab); Pem pooled (pembrolizumab 2 mg /kg, or 10 mg /kg).

Table S13. SUCRA values (ranks) for OS efficacy in the PD-L1-positive group and its subgroups by histology (non-squamous or squamous) (four RCTs of CheckMate057, CheckMate017, OAK, KEYNOTE-010 were included).

	OS	OS non-sq	OS sq
Doc	0.2 (5)	0.0 (3)	5.9 (3)
Niv	76.5 (2)	81.3 (1)	76.9 (1)
Atz	41.9 (4)	NE	NE
Pem2	49.6 (3)	NE	NE
Pem10	81.8 (1)	NE	NE
Pem (pooled)	NE	68.7 (2)	67.2 (2)

The surface under the cumulative ranking curve (SUCRA) value (rank) for the efficacy in terms of overall survival (OS) in patients with programmed cell death ligand 1 (PD-L1) positive (≥1 %) refractory or relapsed advanced NSCLC and subgroup, based on histological type (non-squamous or squamous). Data are listed as SUCRA values with (rank). Doc (docetaxel); Niv (nivolumab); Atz, (atezolizumab); Pem2 (pembrolizumab 2 mg /kg); Pem10 (pembrolizumab 10 mg /kg); Pem pooled; (pembrolizumab 2 mg /kg, or 10 mg /kg).
